# A Rare Case of COVID-19-Induced Chronic Demyelinating Polyneuropathy

**DOI:** 10.7759/cureus.25165

**Published:** 2022-05-20

**Authors:** Dhaval Patel, Gaurav Mandal, Lawrence Chukwueke, Kenneth Woods

**Affiliations:** 1 Internal Medicine, Trinity West Medical Center, Steubenville, USA; 2 Infectious Disease, Trinity West Medical Center, Steubenville, USA

**Keywords:** chronic autoimmune demyelinating polyneuropathy (cidp), critical illness myopathy, guillain barre’s syndrome (gbs), weakness in the icu, acute motor axonal neuropathy, intravenous immunoglobulins (ivig), covid-19

## Abstract

Chronic autoimmune demyelinating polyneuropathy (CIDP) is a rare autoimmune disorder in which the body's immune system attacks the myelin sheaths. Myelin sheaths are the fatty insulation covering and protecting the nerves, and damage to these can lead to neurological symptoms like numbness, tingling, and weakness. CIDP is a chronic disease in the Guillain-Barré syndrome spectrum. Numerous case reports of autoimmune diseases linked to coronavirus disease 2019 (COVID-19) have been seen since the onset of COVID-19 pandemic. We present one such challenging case of COVID-19-induced CIDP.

## Introduction

Coronavirus disease 2019 (COVID-19), caused by severe acute respiratory syndrome coronavirus 2 (SARS-CoV-2), a ribonucleic acid virus, has become a significant public health concern since December 2019, which later evolved into a pandemic [1]. Although SARS-CoV-2 most commonly affects the respiratory, cardiovascular, and gastrointestinal systems, it also has neuroinvasive capabilities. However, the neurological complications from COVID-19 are often less suspected due to their comparatively lower incidences [1,2]. Although anosmia and ageusia are more common neurological symptoms of COVID-19, severe complications such as encephalopathy, cerebrovascular disease, seizures, and even acute inflammatory demyelinating polyneuropathies have been reported [1,3-5]. Also, there is evidence that COVID-19 was the etiology of exacerbation of chronic inflammatory demyelinating polyneuropathy (CIDP) in a case report [6]. We came across a patient who developed CIDP after contracting the SARS-CoV-2 infection while admitted to the intensive care unit.

## Case presentation

A 69-year-old female with a history of type 2 diabetes mellitus, heart failure with reduced ejection fraction, hypertension, and hypothyroidism presented to the hospital with a three-week history of intractable watery diarrhea, nausea, generalized weakness, and myalgias. She also had mild shortness of breath and was noted to be hypoxic, requiring oxygen support.

She was diagnosed with COVID-19 pneumonia and was admitted for sepsis and acute hypoxic and hypoxemic respiratory failure. A 10-day course of 6 mg dexamethasone was initiated to modulate inflammation-mediated lung injury [7]. However, her respiratory status progressively worsened, developing into acute respiratory distress syndrome. She became encephalopathic and could not protect her airway. Mechanical ventilation was required on day six after a witnessed aspiration event and respiratory failure. Over the next three weeks, attempts to liberate the patient from the ventilator were ineffective, and she subsequently required a tracheostomy on day 26. The patient was gradually weaned off of sedation. She was noted to have very limited phonation, which was not present on admission, but she was able to express her symptoms by nodding her head and was following commands. She was noted to have generalized weakness and endorsed persistent pain in the upper extremities over the next few days. On day 34, with an improvement of her mentation, it was possible to test for muscle strengths in this left-hand dominant patient which revealed 2/5 strength in the left upper extremity (LUE) and 1/5 in the remaining limbs and absent reflexes. Myositis was ruled out based on the normal creatine phosphokinase level. A magnetic resonance imaging of the brain showed no evidence of infarction or neoplasm. Doppler ultrasound of the extremities was obtained for concerns of persistent pain in the extremities, which also ruled out deep vein thrombosis. On day 40, electromyography was done, which demonstrated severe axonal neuropathy with demyelinating components involving lower, upper, and cervical nerves. Given the involvement of cervical nerves and acute denervation in multiple nerve roots, findings were suggestive of CIDP. Intravenous immunoglobulin (IVIg) was started. Cerebrospinal fluid (CSF) analysis, as mentioned below, was consistent with cytoalbuminologic dissociation. Over the next five days, the patient gained movements in her left upper extremity with a strength of 4+/5 and only minimal improvements in the movements in her right forearm, right hand, right fingers, and bilateral feet and toes. She continued to have paresthesia and tenderness in all her limbs post-IVIg, which partially responded to gabapentin. Guillain-Barré syndrome (GBS) was also on the differential, but with the continuous progression of symptoms beyond four weeks and the EMG report of nerve root demyelination, the clinical picture was consistent primarily with CIDP. Her diagnosis was challenged by prolonged intubation, as well as multiple differentials such as ICU neuropathy and myopathy, profound debility, and a non-verbal state. A few of the pertinent labs are mentioned in Table 1.

**Table 1:** Pertinent labs CSF: cerebrospinal fluid, WBC: white blood cell count, RBC: red blood cell count, TSH: thyroid-stimulating hormone, CRP: C-reactive protein, NMDA rec Ab: N-methyl-D-aspartate receptor antibody.

Test	Result	Reference range
CSF		
WBC	0/µl	0-5/µl
RBC	1/µl	0/µl
Protein	172 mg/dl	15-60 mg/dl
Glucose	79 mg/dl	40-70 mg/dl
A1c	9.8%	3.8-5.6%
Creatinine	2.9 mg/dl	0.5-1.2 mg/dl
Calcium	8.1 mg/dl	8.5-10.1 mg/dl
TSH	2.5 µIU/ml	0.3-3.7 µIU/ml
Hemoglobin	7.8 g/dl	12-16 g/dl
Hepatitis Bs Antigen	Non-reactive	Non-reactive
CRP	33 mg/L	<3 mg/L
NMDA rec Ab	<1:1	<1:1

## Discussion

CIDP is a rare immune-mediated neurological disorder in which the immune system causes aberrant demyelination and axonal damage to peripheral nerves leading to progressive weakness and impaired sensory function in the legs and arms [8]. CIDP and GBS exist on a spectrum with largely the same etiologies, clinical features, and laboratory findings, although the antecedent event is usually less clear in CIDP [9].

COVID-19 has been deemed as the etiology of fulminant exacerbation of CIDP in a case report previously [6]. However, this is our first encounter with COVID-19-induced CIDP. In addition, the causation of polyneuropathy associated with GBS from COVID-19 has been well established so far [4,5]. This presents an additional challenge because while steroids are a mainstay of treatment for COVID-19, they have no known benefit in Guillain-Barré syndrome and may slow recovery [10]. As such, any polyneuropathy suspected due to COVID-19 must be carefully differentiated before treatment decisions are made. However, the diagnosis and management of these neurological manifestations are still a challenge, and they often go unnoticed due to patients' poor respiratory status requiring mechanical ventilation. In order to improve the diagnostic accuracy and management of our patient, we could have obtained serological markers like antibodies to neurofascin-155, neurofascin-140/186, contactin-1, and contactin-associated protein 1 associated with CIDP [11]. However, this remains the limitation of our case as these tests are send-outs in a community hospital like ours, which could take days to be reported and could, in turn, delay treatment.

Early diagnosis and treatment are of paramount importance as the physical and mental impacts are tremendous. According to one study, 17% and 74% of patients with CIDP reported severe pain and severe fatigue, respectively [12]. Another study reported impairments in daily activities like unzipping, using a knife and fork, and requiring some support for outdoor activities like walking and standing [13]. This case illustrates diagnostic complexity and poor response to treatment due to prolonged ICU stay leading to ICU myopathy and deconditioning. This case also highlights the importance of thorough neurological examination in patients like ours who are otherwise unable to voice their complaints.

## Conclusions

Our case represents a relatively new association of CIDP with SARS-CoV-2 infection. While it highlights an essential complication of COVID-19 that should be kept in mind while examining patients, this case also reminds us that there are many associations about this virus that we have yet to discover fully. It also highlights the importance of maintaining an open and inquisitive mind while treating patients with this disease, as the morbidity and mortality could be very significant.
